# Regulation of the *Pax6 *: *Pax6(5a) *mRNA ratio in the developing mammalian brain

**DOI:** 10.1186/1471-213X-5-13

**Published:** 2005-07-19

**Authors:** Jeni Pinson, John O Mason, T Ian Simpson, David J Price

**Affiliations:** 1 Biomedical Sciences, Hugh Robson Building, University of Edinburgh, George Square, Edinburgh, EH8 9XD, UK

## Abstract

**Background:**

Early in mammalian brain development cell proliferation generates a population of progenitor cells whose subsequent divisions produce increasing numbers of postmitotic neurons. Pax6 affects both processes and it has been suggested that this changing role is due at least in part to changes in the relative concentrations of its two main isoforms, (i) Pax6 and (ii) Pax6(5a), created by insertion of a 42 bp exon (exon 5a) into one of the two DNA-binding domains. Crucially, however, no previous study has determined whether the ratio between *Pax6 *and *Pax6(5a) *transcripts alters during mammalian neurogenesis *in vivo*.

**Results:**

Using RNase protection assays, we show that *Pax6 *transcripts are 6–10 times more prevalent than *Pax6(5a) *transcripts early in neurogenesis in the murine telencephalon, diencephalon and hindbrain and that the ratio later falls significantly to about 3:1 in these regions.

**Conclusion:**

These changes *in vivo *are similar in magnitude to those shown previously to alter target gene activity *in vitro *and might, therefore, allow the single mammalian *Pax6 *gene to carry out different functions at different times in mammalian brain development.

## Background

**Figure 1:**
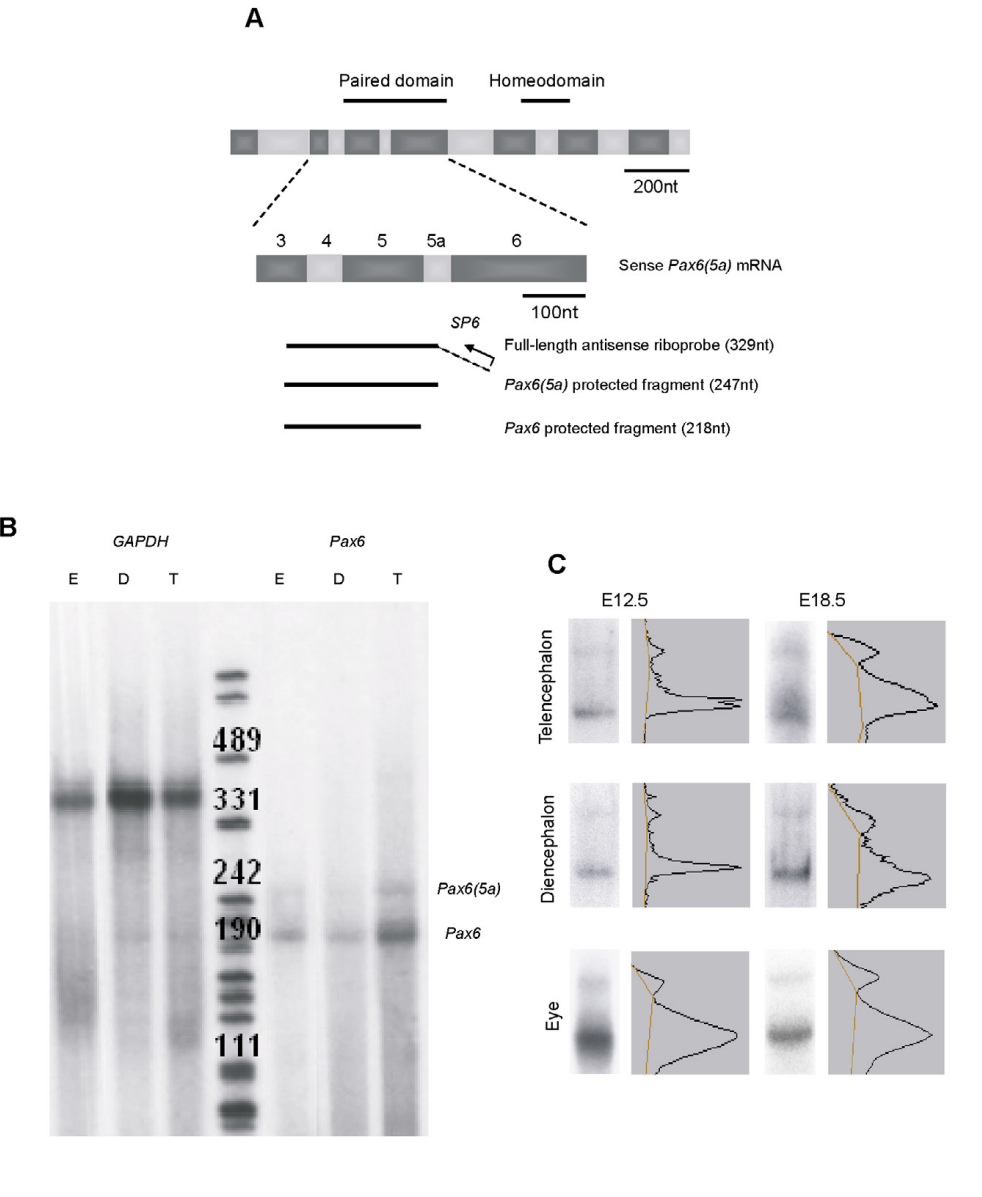
Detection of *Pax6 *and *Pax6(5a)*. (A) Riboprobe design. The ssRNA antisense riboprobe used to detect both *Pax6 *and *Pax6(5a) *spans the *Pax6 *mRNA from exon 3 to exon 5a. Two protected fragments are produced, *Pax6 *(218nt) and *Pax6(5a) *(247nt). The PD- and HD-coding regions are represented above the *Pax6 *mRNA sequence, which is shown to scale. Grey and black bars are exons. Broken line shows part of the riboprobe derived from the vector. (B) Example of an RNase protection assay showing *GAPDH *and *Pax6 *signal detected in total RNA from E12.5 eye (E), diencephalon (D) and telencephalon (T). 1 μg total RNA was used in each sample. Central lane is a DNA ladder, which allows approximate sizing of bands (fragment sizes are indicated above bands). (C) Quantification of RNase protection assays. *Pax6 *(lower band) and *Pax6(5a) *(upper band) in the telencephalon, diencephalon and eye at E12.5 and E18.5. Gel images and densitometric traces of bands are shown. Level of background estimated using the rolling disk method (Quantity One software, Biorad) is indicated on each trace.

*Pax6 *is expressed in the developing eye and brain, where it affects both progenitor cell production and neuronal differentiation [1-5]. The Pax6 protein contains two DNA binding domains, a paired domain (PD) and a paired-type homeodomain (HD) (Fig. 1A). The PD consists of two separate helix-turn-helix motifs, termed PAI and RED, which act on different target sequences [6]. The best characterised *Pax6 *alternative splicing event, involving the insertion of a 42 bp exon (exon 5a) into the PAI subdomain of the PD, results in two major Pax6 isoforms [Pax6 and Pax6(5a)] with different DNA-binding properties. *In vitro *studies have shown that the PAI subdomain of Pax6 binds preferentially to a consensus sequence (P6CON) [7] but that PAI subdomain disruption in Pax6(5a) allows the RED subdomain to bind an alternative sequence (5aCON) [6].

Pax6 affects early progenitor cell proliferation and later neuronal differentiation in the developing brain [4,5]. This changing role might be due at least in part to a shift in the relative concentrations of Pax6 and Pax6(5a) during development but there has been no previous report of such alterations during neurogenesis in the brain *in vivo*. We anticipated that even relatively small changes in the Pax6 : Pax6(5a) ratio might be important since stronger effects on gene activity *via *P6CON and 5aCON are observed if *Pax6 *and *Pax6(5a) *are introduced into cultured cell lines at ratios of 1:1 or 8:1 than at ratios of 2:1, 4:1 or 16:1 [8]. We selected a direct method for quantification of the ratio (RNase protection assays) and carried out multiple assays so as to obtain statistically analysable data on key Pax6-expressing brain tissues at a range of ages throughout neurogenesis. We found changes in the ratio similar in magnitude to those shown previously to alter target gene activity *in vitro*.

## Results and Discussion

RNase protection assays were carried out on tissues dissected from wild type mouse embryos aged E12.5-E18.5 and quantified by densitometry (Fig. 1). The *Pax6 *: *Pax6(5a) *ratio varied throughout neurogenesis in the telencephalon, diencephalon and hindbrain, following a similar pattern in each (Table 1). In the telencephalon, the ratio was about 6:1 early in neurogenesis, at E12.5, but was significantly lower (about 2:1) at each subsequent age (p < 0.05–p < 0.01; Table 1). In the diencephalon and hindbrain, ratios were about 8:1 and 10:1 at E12.5 but were lower (about 2:1 to 4:1) from E14.5-E18.5 (Table 1); these decreases were statistically significant when data from all three age groups were combined (p < 0.05 for both tissues). Combining results from the telencephalon, diencephalon and hindbrain showed a significant fall in the *Pax6 *: *Pax6(5a) *ratio between E12.5 and E14.5, from approximately 8:1 to 3:1 (p < 0.05), with a further slight decrease at E16.5 and E18.5 (p < 0.01; Table 1). Data on the *Pax6 *: *Pax6(5a) *ratio in the eye at E12.5-E18.5 did not show the same trend (Table 1). Ratios varied from about 10:1 at E14.5 to about 4:1 at E18.5 but none of the differences were statistically significant. Although we did not detect significant changes in the eye, it is possible that non-synchronised changes in the ratio do occur within its component Pax6-expressing neural and non-neural tissues (cornea, lens and retina). Neither *Pax6 *nor *Pax6(5a) *was detected in samples from the feet.

**Table 1:** Mean *Pax6*: *Pax6(5a) *ratios ± SEMs. "Combined" data are from telencephalon, diencephalon and hindbrain considered together at each age. For each tissue and for combined brain tissues, significant differences between values at E12.5 and values at subsequent ages are shown (unpaired Student's *t*-tests; * = p < 0.05, ** = p < 0.01). Numbers of individual assays are indicated in brackets.

	**E12.5**	**E14.5**	**E16.5**	**E18.5**
**Telencephalon**	6.25 +/- 0.68 (5)	2.12 +/- 0.77** (2)	2.63 +/- 1.52* (3)	2.53 +/- 0.37** (4)
**Diencephalon**	9.72 +/- 3.37 (3)	3.63 +/- 0.63 (3)	1.41 (1)	3.00 +/- 0.63 (2)
**Hindbrain**	7.66 +/- 3.68 (4)	3.87 +/- 1.62 (2)	1.66 +/- 0.35 (2)	2.23 +/- 0.12 (3)
**Combined**	7.59 +/- 1.41 (12)	3.27 +/- 0.55* (7)	2.10 +/- 0.72** (6)	2.53 +/- 0.21** (9)
**Eye**	6.81 +/- 2.08 (4)	9.52 +/- 3.64 (3)	5.95 +/- 1.90 (3)	3.55 +/- 1.30 (3)

There is evidence that Pax6 and Pax6(5a) have different functions *in vivo *in both vertebrates [9-11] and invertebrates [12,13]. Studies of *Pax6*- and *Pax6(5a)*-related genes in *Drosophila melanogaster*, *ey/ toy *and *eyg/ toe*, have shown that they promote, respectively, differentiation and proliferation of eye precursor cells [12,13]. Overexpression of Pax6 and Pax6(5a) can alter the expression of different sets of genes in mammals [10,11]. Mammalian brain cells reduce their proliferation in response to overexpression of Pax6 or Pax6(5a) and increase their neurogenesis in response to overexpression of Pax6 [9]. It is possible that a reduction in the *Pax6 *: *Pax6(5a) *mRNA ratio from E14.5 is involved in programming progenitor cells to initiate processes that occur later in embryogenesis. Such processes are potentially numerous; they might, for example, include the transition from predominantly neurogenesis to the major phase of gliogenesis or the development of specific sets of later-generated neurones such as the superficial layers of the cerebral cortex.

## Conclusion

We conclude that the *Pax6 *: *Pax6(5a) *ratio falls in the telencephalon, diencephalon and hindbrain during neurogenesis and, moreover, the magnitude of the change is in the range that alters target gene expression *in vitro *[8]. This finding allows the possibility that changes in the relative expression levels of isoforms of a single *Pax6 *gene might result in changes in the functions of this gene in mammalian brain development.

## Methods

For each RNase protection assay, wild-type mice (CD-1) were time-mated [the day of conception was designated embryonic day 0.5 (E0.5)] and killed on E12.5, E14.5, E16.5 or E18.5. The telencephalon, diencephalon, hindbrain, eyes and feet (used as a Pax6 non-expressing control tissue) were dissected from each embryo. Tissues from multiple embryos were combined so as to obtain sufficient material for each assay. Total RNA was isolated from tissue snap-frozen in liquid nitrogen, its integrity was checked by agarose gel electrophoresis and its concentration was measured using a fluorimeter. The riboprobe was designed to span the *Pax6 *mRNA from exon 3 to exon 5a so as to protect *Pax6 *and *Pax6(5a) *mRNAs (Fig. 1A). Oligonucleotides 5'-AAG TGG ACG TAT ATC CCA GTT CTC-3' and 5'-AGC ACC TGG ACT TTT GCA TC-3' were used to amplify sequence from mouse *Pax6 *cDNA. *Pax6 *cDNA was cloned into pCR-BluntII-TOPO and sequenced across the *Pax6 *insert in both directions to identify clones with *Pax6 *sequence in the appropriate orientation to generate antisense probe. The plasmid pTRI-GAPDH (Ambion) was used for synthesis of a murine glyceraldehyde-3-phosphate dehydrogenase (GAPDH) riboprobe. Riboprobe synthesis was carried out using the Maxiscript SP6 *in vitro *transcription kit (Ambion) in the presence of 40 μCi α-^32^P-UTP 800 Ci/mmol (Amersham). Riboprobes were purified using a MicroSpin G25 column (Amersham Biosciences).

RNase protection assays were performed on 1 μg total RNA from E12.5 and E14.5 embryonic tissues, or 4 μg total RNA from E16.5 and E18.5 embryonic tissues, using the Hyb Speed kit (Ambion). The quality the undigested full-length riboprobe was examined and that of all total RNA samples was assessed using a *GAPDH *riboprobe (e.g. Fig. 1B). Products were resolved on 6% polyacrylamide, 8 M urea gels that were then fixed (15% methanol, 5% acetic acid for 30 minutes), dried and exposed to film with an intensifying screen. Where *GAPDH *signal was weak, this was a sign that protein or DNA contamination had led to inaccurate RNA quantification, and these samples were excluded from subsequent analyses. Gel bands were quantitated using a GS-710 densitometer and the Quantity One software package (BioRad: background subtraction was performed using the rolling disk method [14]; Fig. 1C). Each assay was repeated several times (n values for the number of assays for each tissue at each age are in Table 1).

## Authors' contributions

JP carried out all the RNase protections assays, analysed the results and wrote a first draft of the manuscript. JP, JM, IS and DP all helped conceive and design the study and all worked on and approved the final manuscript.
